# Identifying Distinct Risk Thresholds of Glycated Hemoglobin and Systolic Blood Pressure for Rapid Albuminuria Progression in Type 2 Diabetes From NHANES (1999–2018)

**DOI:** 10.3389/fmed.2022.928825

**Published:** 2022-06-20

**Authors:** Jiahui Xu, Yan Xue, Qingguang Chen, Xu Han, Mengjie Cai, Jing Tian, Shenyi Jin, Hao Lu

**Affiliations:** ^1^Department of Endocrinology, Shuguang Hospital Affiliated to Shanghai University of Traditional Chinese Medicine, Shanghai University of Traditional Chinese Medicine, Shanghai, China; ^2^Laboratory of Cellular Immunity, Shuguang Hospital Affiliated to Shanghai University of Traditional Chinese Medicine, Shanghai University of Traditional Chinese Medicine, Shanghai, China

**Keywords:** risk thresholds, glycated hemoglobin, systolic blood pressure, albuminuria, type 2 diabetes, NHANES

## Abstract

**Background:**

It is widely recognized that glycated hemoglobin (HbA1c) and systolic blood pressure (SBP) are two key risk factors for albuminuria and renal function impairment in patients with type 2 diabetes mellitus (T2DM). Our study aimed to identify the specific numerical relationship of albumin/creatinine ratio (ACR) with HbA1c and SBP among a large population of adults with T2DM.

**Method:**

A total of 8,626 patients with T2DM were included in the data analysis from the National Health and Nutrition Examination Surveys (NHANES) (1999-2018). The multiple linear regressions were used to examine the associations of ACR with HbA1c and SBP. Generalized additive models with smooth functions were performed to identify the non-linear relations between variables and interactions were also tested.

**Results:**

Significantly threshold effects were observed between ACR and HbA1c or SBP after multivariable adjustment, with the risk threshold values HbA1c = 6.4% and SBP = 127 mmHg, respectively. Once above thresholds were exceeded, the lnACR increased dramatically with higher levels of HbA1c (β = 0.23, 95 CI%:0.14, 0.32, *P* < 0.001) and SBP (β = 0.03, 95 CI%:0.03, 0.04, *P* < 0.001). Subgroup analysis showed high protein diet was related to higher ACR. In addition, a higher risk of ACR progression was observed in central obesity participants with HbA1C ≥ 6.4% or hyperuricemia participants with SBP ≥ 127 mmHg among patients withT2DM.

**Conclusion:**

We identified thresholds of HbA1c and SBP to stratify patients with T2DM through rapid albuminuria progression. These might provide a clinical reference value for preventing and controlling diabetes kidney disease.

## Introduction

Progression of albuminuria in diabetic patients is associated with impaired renal function and indicative of an increased risk of cardiovascular disease (CVD). Studies have demonstrated that in patients with type 2 diabetes mellitus (T2DM), microalbuminuria is considered an early marker for renal function decline, and elevated albuminuria was consistently correlated with the risk of end-stage kidney disease (1, 2). In addition, as an indicator of the systemic endothelial dysfunction response (3), increased albuminuria also predicts higher risks of myocardial infarction, heart failure, stroke, and cardiac death (4–6). Therefore, it is essential to assess albuminuria in diabetic patients. Since the albumin/creatinine ratio (ACR) is a reliable and sensitive index reflecting early kidney damage as well as relatively stable and convenient, ACR is commonly used to estimate the degree of urinary protein excretion clinically (7).

Although various risk factors could affect the development of albuminuria, abundant studies have confirmed that raised blood pressure and dysglycaemia are two critical risk factors for albuminuria (8–11). Cumulative evidence emphasizes that control of glycated hemoglobin (HbA1c) and systolic blood pressure (SBP) are significant in decreased ACR for both T2DM and Diabetic kidney disease (DKD) patients (12, 13). Previously, a study identified a 5.5% HbA1c level as the risk threshold for albuminuria prevalence in a large Chinese population over the age of 40 (14). Another study found a significantly increased risk of albuminuria in participants with HbA1c ≥ 7% compared with the normal urinary protein population. The above results remained stable in diabetic and non-diabetic populations (15). This might suggest a threshold effect between HbA1c and ACR levels, but a lack of large-scale population studies targeting patients with T2DM. In addition, the studies on the risk relationship between SBP and ACR have also been extensively reported. A meta-analysis included 31 cohorts in the world and demonstrated that each 20 mmHg increase in SBP was associated with a 1.5-fold higher prevalence of albuminuria (ACR ≥ 30 mg/g) in diabetes (11). It was also reported that only SBP ≤ 120 mmHg was associated with the lowest risk of new-onset microalbuminuria (16). However, almost all the above studies use a recommended cut-off point of 30 mg/g for ACR to explore the effects of HbA1c and SBP on the risk of albuminuria. Notably, A cohort study with an up to11-year follow-up period found that protein excretion levels, even with normal at baseline, are pronouncedly associated with increased mortality risk from CVD (17). A recent study also confirmed that a normal ACR range (≤30 mg/g) was related to left ventricular hypertrophy in patients with T2DM (18). This suggested that the specific numerical changes of ACR and the risk thresholds might not be fully reflected when we simply treated ACR as a categorical variable with a 30 mg/g cut-off.

Thus, in this study, we treated ACR as a continuous variable and included a large-scale T2DM population to explore the specific association of ACR with SBP and HbA1c simultaneously.

## Research Design and Methods

### Study Population

**FIGURE 1:**
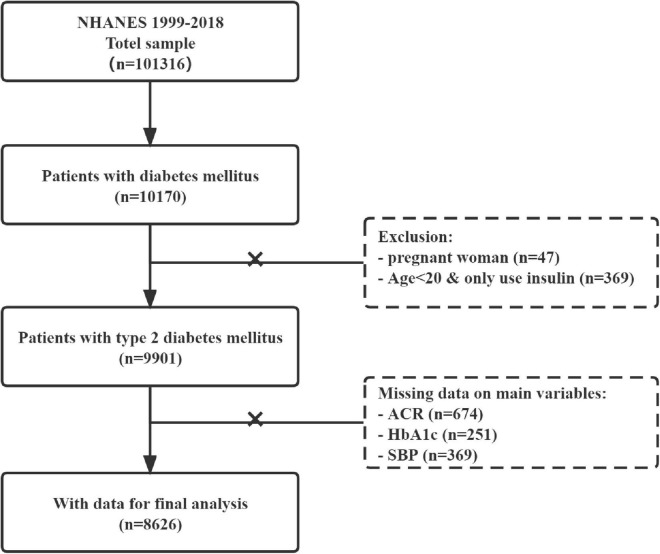
Flow diagram of study population selection.

In this cross-sectional study, we merged all the National Health and Nutrition Examination Surveys (NHANES) data from 1999 to 2018. A total of 10,170 diabetes patients were identified according to the definition. We further identified 9,901 patients with T2DM after excluding pregnant woman (*n* = 47) and possible individuals with type 1 diabetes (*n* = 369). All the missing data for key variables, including ACR (*n* = 674), HbA1c (*n* = 251), and SBP (*n* = 369), were removed from the dataset. Eventually, 8,626 patients with T2DM were included in the final data analyses. The flow chart of the included study population is shown in Figure 1.

### Definition of Diabetes

Diabetes was defined if each condition was satisfied in the following items according to the recent American Diabetes Association (ADA) recommendation (19): (1) Previous diagnosis of type 2 diabetes by doctors (2) Fasting blood glucose levels greater than or equal to 7.0 mmol/L (126 mg/dL) (3) Postprandial 2 h plasma glucose levels greater than or equal to 11.1 mmol/L (200 mg/dL) after a standard 75-g oral glucose tolerance test (4) HbA1c levels were 6.5% (48 mmol/mol) or higher (5) The use of insulin or hypoglycemic drugs. Possible type 1 diabetes patients were defined as those aged <20 years who were only treated with insulin (20).

### Measurement of Main Variables

The albumin/creatinine ratio was calculated from random urine spot collections and reported as mg/g. Therein, the fluorescent immunoassay was employed to measure human urinary albumin and proved to be a reliable and accurate method. The Jaffé method was used to measure urine creatinine (period 1999–2007), and then the enzymatic method was used (period 2008–2018). HbA1c was tested by high-performance liquid chromatography after collecting venous whole blood specimens in EDTA. Above detection, operations were completed in the laboratory at the University of Minnesota and Columbia. More information on sample collection, transport, and processing was available in the NHANES manual. Blood pressure (BP) was measured by trained survey personnel when participants had rested for at least 5 min in a seated position. BP values included in the final analysis were the average of the three consecutive readings obtained with a standard mercury sphygmomanometer (interrupted or incomplete reading was replaced with fourth BP reading). Pulse pressure (PP) was calculated as systolic minus diastolic pressure.

### Definition of Other Variables

Among the demographic parameters, marital status was divided into living with a partner and live without a partner; education level was divided into less than high school, high school, and more than high school. Cigarette smoking status was classified as current smokers (average cigarettes ≥ 1/day), past smokers (average cigarettes <1/day or ≥ 100-lifetime cigarettes but currently non-smoking), and never smokers (<100-lifetime cigarettes or never smoked). The consumption of alcohol was divided into two categories according to whether respondents had at least 12 drinks a year (21). Dietary intake, including dietary protein, sodium intake, and potassium intake, was assessed by two 24 h recalls (one in person and another by telephone 3–10 days later). Meanwhile, the sodium/potassium (Na/K) ratio was calculated for further analysis since the Na/K ratio was proved to have a stronger association with BP than either electrolyte examined alone (22). When obesity indicators were determined as categorical variables, body mass index (BMI, kg/m2) was grouped into normal weight (<25), overweight (≥25, <30), and obese (≥30). A waist circumference ≥102 cm for men and ≥88 cm for women indicates central obesity (23). Diabetes duration was analyzed as a categorical variable with <5 years, ≥5, <10 years, ≥10 years, and not recorded (missing data). The homeostasis model assessment of insulin resistance (HOMA-IR) was calculated with the formula [fasting glucose (mmol/L) × fasting insulin (μU/L)]/22.5. Estimate glomerular filtration rate (eGFR) was calculated based on the chronic kidney disease epidemiology collaboration (CKD-EPI) formula (24).

### Statistical Processing and Analyses

To minimize bias brought by missing data, missing categorical covariates were coded as a separate category as appropriate, and missing continuous covariates were replaced by group means. In addition, allowing for the complex sampling design, all analyses were performed incorporating the sampling weights according to NHANES guidelines (25). First, new multi-year sample weights were calculated using ten survey cycles (using 4-year weights when combining the 1999–2000 and 2001–2002 survey cycles). Then the weights of the smallest subpopulation that includes all the variables were selected for final analysis. Finally, to estimate variance, Taylor series linearization was applied, and all estimates were weighted.

In the baseline data assessment, the study population was stratified into four groups according to ACR quartiles. Continuous variables are presented as means ± *SD*s, and categorical variables are reported as frequencies and percentages. ACR was transformed with the natural logarithm function (LnACR) to stabilize variance prior to analysis. Comparison of continuous variables among groups was analyzed by one-way ANOVA or non-parametric test. The counting variables were analyzed by the chi-square test. Multiple linear regression models were performed to estimate the crude association of ACR with HbA1c and SBP after varying degrees of covariates adjustments. The fully adjusted model included covariates for age, sex, education level, marital status, smoking, alcohol consumption, diabetes duration, BMI, waist circumference, fasting plasma glucose (FPG), diastolic blood pressure (DBP), triglyceride (TG), uric acid (UA), eGFR, SBP/HbA1c, and dietary protein. Covariates listed above were screened based on their regression coefficients relative to ACR with a *P*-value of less than 0.1 (26). There was no multicollinearity effect among the covariates (variance inflation factor (VIF) = 1–4.7). It should be noted that PP was not included as a covariate because of strong collinearity among PP and SBP (VIF > 10). Also, SBP was more positively correlated to ACR than PP, which was consistent with previous studies (27, 28) and demonstrated a stronger relationship between SBP and risk of ACR. Generalized additive models with smooth functions captured the non-linear relationships of ACR with HbA1c and SBP. Then, the threshold levels of HbA1c and SBP were determined using a recursive approach. Likelihood ratio tests were used to assess the difference in fit between the one-line linear regression model with the two-piecewise linear regression model, and *P* < 0.05 was considered significant. Finally, interaction tests were performed between subgroups. Data were analyzed using statistical packages R (The R Foundation; version 3.4.3)^1^ and EmpowerStats software (X&Y Solutions, Inc., Boston, MA, United States).^2^

## Results

### Study Population Characteristics

The detailed clinical characteristics of the 8,626 patients with T2DM included in our study were listed in Table 1. When the study population was stratified into four groups according to ACR quartiles. Age, the percentage of participants living with a partner, proportion of participants with an educational level less than high school, the number of current smokers, the proportions of participants with a long diabetes duration (≥10 years), the proportions of participants taking antihypertensive medication, FPG, HbA1c, TG, SBP, PP, and UA levels all showed increased tendency between the four groups with elevated ACR level. BMI was significantly different across groups after being transformed into a categorical variable. No significant differences were observed in Na/K ratio, waist circumference, DBP, total cholesterol (TC), and alanine aminotransferase (ALT).

**TABLE 1:** The clinical characteristics of enrolled participants were stratified by albumin/creatinine ratio (ACR) quartiles.

Characteristic	ACR (mg/g)				*p*-value
	Q1 (<6.58) *n* = 2,154	Q2 (6.58 - 12.62) *n* = 2,158	Q3 (12.62 - 40.46) *n* = 2,157	Q4 (≥40.46) *n* = 2,157	
Age (years)	57.56 ± 13.59	60.76 ± 13.56	62.15 ± 14.09	64.08 ± 13.42	<0.001
Sex					<0.001
Male	1198 (55.62%)	1022 (47.36%)	1032 (47.84%)	1237 (57.35%)	
Female	956 (44.38%)	1136 (52.64%)	1125(52.16%)	920 (42.65%)	
Race					<0.001
Mexican American	379 (17.60%)	420 (19.46%)	439 (20.35%)	502 (23.27%)	
Other Hispanic	196 (9.10%)	207 (9.59%)	219 (10.15%)	195 (9.04%)	
Non-hispanic White	814 (37.79%)	836 (38.74%)	830 (38.48%)	749 (34.72%)	
Non-hispanic black	560 (26.00%)	466 (21.59%)	460 (21.33%)	530 (24.57%)	
Other race	205 (9.52%)	229 (10.61%)	209 (9.69%)	181 (8.39%)	
Marital status					<0.001
Living with partner	1399 (64.95%)	1296 (60.06%)	1264 (58.60%)	1203 (55.77%)	
Living without partner	740 (34.35%)	847 (39.25%)	882 (40.89%)	937 (43.44%)	
Not recorded	15 (0.70%)	15 (0.70%)	11 (0.51%)	17 (0.79%)	
Education level					<0.001
Less than high school	664 (30.83%)	754 (34.94%)	791 (36.67%)	946 (43.86%)	
High school	498 (23.12%)	516 (23.91%)	492 (22.81%)	460 (21.33%)	
More than high school	992 (46.05%)	888 (41.15%)	874 (40.52%)	751 (34.82%)	
Smoking					<0.001
Current	328 (15.23%)	335 (15.52)	314 (14.56)	346 (16.04)	
Past	700 (32.50)	720 (33.36%)	745 (34.54%)	821 (38.06%)	
Never	1126 (52.27%)	1103 (51.11%)	1098 (50.90%)	990 (45.90%)	
Alcohol consumption					<0.001
Yes	1306 (60.63%)	1178 (54.59%)	1153 (53.45%)	1199 (55.59%)	
No	707 (32.82%)	842 (39.02%)	856 (39.68%)	806 (37.37%)	
Not recorded	141 (6.55%)	138 (6.39%)	148 (6.86%)	152 (7.05%)	
Dietary protein (g/d)	79.28 ± 35.07	74.56 ± 32.40	73.88 ± 32.40	73.19 ± 33.52	<0.001
Sodium intake (mg/d)	3300.99 ± 1562.04	3130.04 ± 1446.84	3122.00 ± 1407.22	3035.04 ± 1459.83	<0.001
Potassium intake (mg/d)	2597.78 ± 1050.19	2516.54 ± 1053.73	2489.65 ± 1062.69	2382.29 ± 1006.80	<0.001
Na/K ratio	1.34 ± 0.50	1.31 ± 0.50	1.33 ± 0.50	1.34 ± 0.52	0.172
Diabetes duration (years)					<0.001
<5	364 (16.90%)	339 (15.71%)	328 (15.21%)	227 (10.52%)	
≥5, <10	217 (10.07%)	275 (12.74%)	253 (11.73%)	233 (10.80%)	
≥10	418 (19.41%)	461 (21.36%)	516 (23.92%)	803 (37.23%)	
Not recorded	1155 (53.62%)	1083 (50.19%)	1060 (49.14%)	894 (41.45%)	
BMI (kg/m^2^)	32.03 ± 6.98	31.85 ± 7.23	31.68 ± 7.25	31.60 ± 7.04	0.141
BMI (kg/m^2^)					0.022
<25	268 (12.44%)	322 (14.92%)	326 (15.11%)	343 (15.90%)	
≥25, <30	683 (31.71%)	644 (29.84%)	668 (30.97%)	616 (28.56%)	
≥ 30	1203 (55.85%)	1192 (55.24%)	1163 (53.92%)	1198 (55.54%)	
Waist circumference (cm)	107.55 ± 15.13	107.41 ± 15.26	107.58 ± 15.62	108.35 ± 15.22	0.121
Waist circumference (cm)					0.052
<102(male), < 88(female)	525 (24.37%)	459 (21.27%)	461 (21.37%)	483 (22.39%)	
≥102(male), ≥ 88(female)	1629 (75.63%)	1699 (78.73%)	1696 (78.63%)	1674 (77.61%)	
HOMA-IR					<0.001
Lower group	481 (22.33%)	453 (20.99%)	427 (19.80%)	387 (17.94%)	
Higher group	425 (19.73%)	472 (21.87%)	452 (20.96%)	400 (18.54%)	
Not recorded	1248 (57.94%)	1233 (57.14%)	1278 (59.25%)	1370 (63.51%)	
FPG (mmol/L)	7.62 ± 3.01	8.04 ± 3.27	8.79 ± 4.02	9.46 ± 4.59	<0.001
HbA1c (%)	6.69 ± 1.36	6.95 ± 1.54	7.30 ± 1.77	7.75 ± 2.07	<0.001
SBP (mmHg)	125.26 ± 15.49	129.13 ± 17.38	134.24 ± 20.34	141.95 ± 23.63	<0.001
DBP (mmHg)	69.15 ± 11.96	69.30 ± 12.99	69.45 ± 13.81	69.98 ± 15.25	0.225
PP (mmHg)	56.11 ± 17.27	59.83 ± 18.62	64.79 ± 21.22	71.97 ± 24.71	<0.001
TC(mmol/L)	4.91 ± 1.15	4.93 ± 1.12	4.97 ± 1.24	4.99 ± 1.36	0.757
TG (mmol/L)	2.03 ± 1.64	2.05 ± 1.75	2.31 ± 2.08	2.39 ± 2.41	<0.001
HDL-C (mmol/L)	1.25 ± 0.36	1.28 ± 0.39	1.24 ± 0.37	1.24 ± 0.40	0.009
ALT (U/L)	27.51 ± 18.99	27.16 ± 20.55	27.22 ± 20.29	26.54 ± 38.09	0.652
AST (U/L)	26.44 ± 15.26	26.48 ± 22.14	26.51 ± 15.23	26.46 ± 21.53	0.002
Albumin (G/L)	41.48 ± 3.21	41.73 ± 3.18	41.68 ± 3.23	40.61 ± 3.80	<0.001
UA(umol/L)	343.55 ± 83.88	333.48 ± 87.50	335.04 ± 95.04	357.41 ± 101.19	<0.001
Scr (umol/L)	82.33 ± 23.14	78.18 ± 24.10	80.11 ± 30.13	105.23 ± 81.42	<0.001
eGFR (ml/min/1.73 m^2^)	83.98 ± 23.92	86.71 ± 27.37	86.09 ± 29.60	75.84 ± 35.53	<0.001
**Taking medication**					
ACEI/ARB	913 (42.39%)	991 (45.92%)	1034 (47.94%)	1118 (51.83%)	<0.001
SGLT-2	8 (0.37%)	22 (1.02%)	15 (0.70%)	9 (0.42%)	0.026

*ACR, albumin/creatinine ratio; Na/K ratio, sodium/potassium ratio; BMI, body mass index; HOMA-IR, homeostasis model assessment of insulin resistance; FPG, fasting plasma glucose; HbA1c, glycated hemoglobin; SBP, systolic blood pressure; DBP, diastolic blood pressure; PP, pulse pressure; TC, total cholesterol; TG, triglyceride; HDL-C, high-density lipoprotein cholesterol; ALT, alanine aminotransferase; AST, aspartate aminotransferase; UA, uric acid; Scr, serum creatinine; eGFR, estimated glomerular filtration rate; ACEI, angiotensin-converting enzyme inhibitor; ARB, angiotensin receptor blocker; SGLT-2, sodium-glucose cotransporter 2. Data are present as n (%) or the mean ± standard deviation.*

### Association Between Albumin/Creatinine Ratio and HbA1c or Systolic Blood Pressure

To comprehensively explore the relationship of ACR with HbA1c and SBP, we conducted different linear regression models when the independent variables were both treated as continuous and categorical variables. Increased HbA1c and SBP levels (continuous variable) have consistently shown an association with increased lnACR level (*P* < 0.001) whether in the non-adjusted model, the multivariate-adjusted model I and II (Table 2). HbA1c and SBP were then transformed into categorical variables by fixed intervals. In the fully adjusted multivariable model II, compared with the reference group of HbA1c (HbA1c < 6), no significant elevated lnACR levels were observed in the second HbA1c group (β = 0.05, 95 CI%: −0.02, 0.12, *P* = 0.156), but the positive association became statistically significant from the third group (β = 0.18, 95 CI%:0.09, 0.27, *P* < 0.001) to highest HbA1c group (β = 0.81, 95 CI%:0.68, 0.94, *P* < 0.001) (Table 2). The Changes in SBP also displayed similar trends. Compared to the first group of SBP in multiple linear regression models, only the second group of SBP levels had no relationship with an increased level of lnACR (β = 0.02, 95 CI%: −0.09, 0.12, *P* = 0.771) (Table 2). The above results suggested that the positive linear relationships were not always consistent between ACR and HbA1c or SBP. Potential threshold effects might exist in the lower groups of HbA1c and SBP.

**TABLE 2:** The relationship between ACR and HbA1c or SBP using linear regression analysis.

	LnACR (mg/g)						
	N	Non-adjusted model		Multivariate-adjusted model I		Multivariate-adjusted model II	
		β (95CI)	*p*-value	β (95CI)	*p*-value	β (95CI)	*p*-value
HbA1C (%) (continuous variable)	8626	0.21 (0.19, 0.22)	<0.001	0.19 (0.17, 0.21)	<0.001	0.16 (0.13, 0.18)	<0.001
**HbA1C (%)** **(categorical variable)**							
<6.0	2256	reference		reference		reference	
6.0–7.0	2851	0.20 (0.13, 0.28)	<0.001	0.08 (0.01, 0.16)	0.030	0.05 (−0.02, 0.12)	0.160
7.0–8.0	1600	0.49 (0.40, 0.58)	<0.001	0.30 (0.21, 0.40)	<0.001	0.19 (0.10, 0.27)	<0.001
8.0–9.0	779	0.64 (0.52, 0.75)	<0.001	0.48 (0.37, 0.60)	<0.001	0.38 (0.26, 0.49)	<0.001
≥9.0	1140	1.08 (0.98, 1.19)	<0.001	1.02 (0.91, 1.12)	<0.001	0.81 (0.68, 0.94)	<0.001
SBP(mmHg) (continuous variable)	8626	0.03 (0.02, 0.03)	<0.001	0.02 (0.02, 0.03)	<0.001	0.02 (0.02, 0.02)	<0.001
**SBP (mmHg)** **(categorical variable)**							
<110	861	reference		reference		reference	
110–120	1487	−0.01 (−0.11, 0.10)	0.912	0.02 (−0.09, 0.12)	0.734	0.02 (−0.09, 0.12)	0.768
120–130	1919	0.21 (0.11, 0.32)	<0.001	0.20 (0.10, 0.30)	<0.001	0.17 (0.06, 0.27)	0.001
130–140	1708	0.37 (0.26, 0.47)	<0.001	0.33 (0.22, 0.44)	<0.001	0.31 (0.20, 0.41)	< 0.001
140–150	1091	0.72 (0.60, 0.84)	<0.001	0.68 (0.56, 0.80)	<0.001	0.65 (0.53, 0.77)	< 0.001
150–160	697	1.09 (0.95, 1.23)	<0.001	1.05 (0.92, 1.19)	<0.001	0.98 (0.84, 1.11)	< 0.001
≥160	863	1.76 (1.63, 1.90)	<0.001	1.70 (1.57, 1.84)	<0.001	1.62 (1.48, 1.75)	< 0.001

*LnACR, ln-transformed albumin/creatinine ratio; HbA1c, glycated hemoglobin; SBP, systolic blood pressure. Multivariate-Adjusted Model I adjusted for: age, sex, marital status, education level, smoking, alcohol consumption, diabetes duration, body mass index (continuous), and waist circumference (continuous). Multivariate-Adjusted Model II adjusted for: age, sex, marital status, education level, smoking, alcohol consumption, diabetes duration, body mass index (continuous), waist circumference (continuous), fasting plasma glucose, glycated hemoglobin/systolic blood pressure, diastolic blood pressure, triglyceride, uric acid, estimated glomerular filtration rate and dietary protein.*

### Non-linearity of Albumin/Creatinine Ratio With HbA1c and Systolic Blood Pressure

**FIGURE 2:**
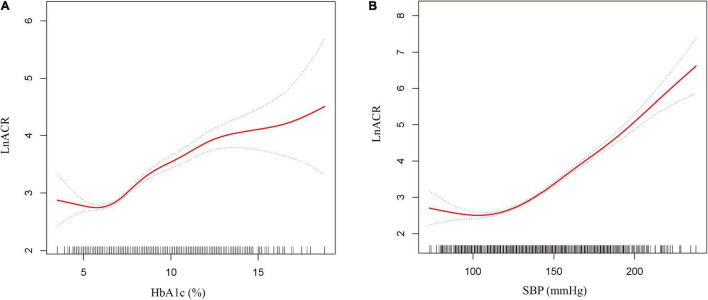
Non-linear relationship between ACR and HbA1c or SBP. **(A)** lnACR with HbA1c **(B)** lnACR with SBP. The solid red line is the fitted curves and the dotted curves are the 95 CI of the fit. All analyses were adjusted for age, sex, marital status, education level, smoking, alcohol consumption, diabetes duration, body mass index (continuous), waist circumference (continuous), fasting plasma glucose, glycated hemoglobin/systolic blood pressure, diastolic blood pressure, triglyceride, uric acid, estimated glomerular filtration rate and dietary protein.

Generalized additive models with smooth functions further revealed the non-linear relationships between lnACR and HbA1c or SBP (Figure 2). Data were fitted with the segmented linear models, and two turning points were determined (HbA1c: 6.4%, SBP: 127 mmHg). The likelihood-ratio tests demonstrated that the two-piecewise linear regression models had a better fit (*P* < 0.001) (Table 3). However, the threshold effect of HbA1c became significant only after adjustment for confounders, while the threshold effect of SBP remained throughout whether or not the confounders were adjusted. After multivariate adjustment in model II, below the thresholds, no significant correlations were observed between lnACR and HbA1c or SBP. Above the thresholds, lnACR was increased significantly with the increment of HbA1c (β = 0.19, 95 CI%:0.16, 0.22, *P* < 0.001) and SBP (β = 0.03, 95 CI%:0.03, 0.04, *P* < 0.001) (Table 3). Notably, the corresponding ACR (mg/g) values for thresholds of HbA1c and SBP were 15.03 (14.44–15.8) and 12.55 (11.94–13.2), respectively, both values being in the normoalbuminuric range (ACR < 30 mg/g).

**TABLE 3:** Threshold effect analysis of HbA1c or SBP on ACR using two-piecewise linear regression.

	LnACR (mg/g)						
	N	Non-adjusted model		Multivariate-adjusted model I		Multivariate-adjusted model II	
				
		β (95CI)	*p*-value	β (95CI)	*p*-value	β (95CI)	*p*-value
**HbA1C (%)**							
<6.4	3184	0.25 (0.14, 0.37)	<0.001	0.07 (−0.05, 0.18)	0.254	0.07 (−0.04, 0.18)	0.258
≥6.4	5442	0.19 (0.17, 0.22)	<0.001	0.21 (0.19, 0.24)	<0.001	0.19 (0.16, 0.22)	<0.001
P for log-likelihood ratio test			0.092		0.002		<0.001
**SBP (mmHg)**							
<127	3760	0.01 (0.00, 0.01)	0.024	0.00 (0.00, 0.01)	0.031	0.00 (0.00, 0.01)	0.051
≥127	4866	0.03 (0.03, 0.04)	<0.001	0.03 (0.03, 0.04)	<0.001	0.03 (0.03, 0.04)	<0.001
P for log-likelihood ratio test			<0.001		<0.001		<0.001

*LnACR, ln-transformed albumin/creatinine ratio; HbA1c, glycated hemoglobin; SBP, systolic blood pressure. Multivariate-Adjusted Model I adjusted for: age, sex, marital status, education level, smoking, alcohol consumption, diabetes duration, body mass index (continuous), and waist circumference (continuous). Multivariate-Adjusted Model II adjusted for: age, sex, marital status, education level, smoking, alcohol consumption, diabetes duration, body mass index (continuous), waist circumference (continuous), fasting plasma glucose, glycated hemoglobin/systolic blood pressure, diastolic blood pressure, triglyceride, uric acid, estimated glomerular filtration rate and dietary protein.*

### Combined Thresholds Analysis and Subgroups Analyses

**FIGURE 3:**
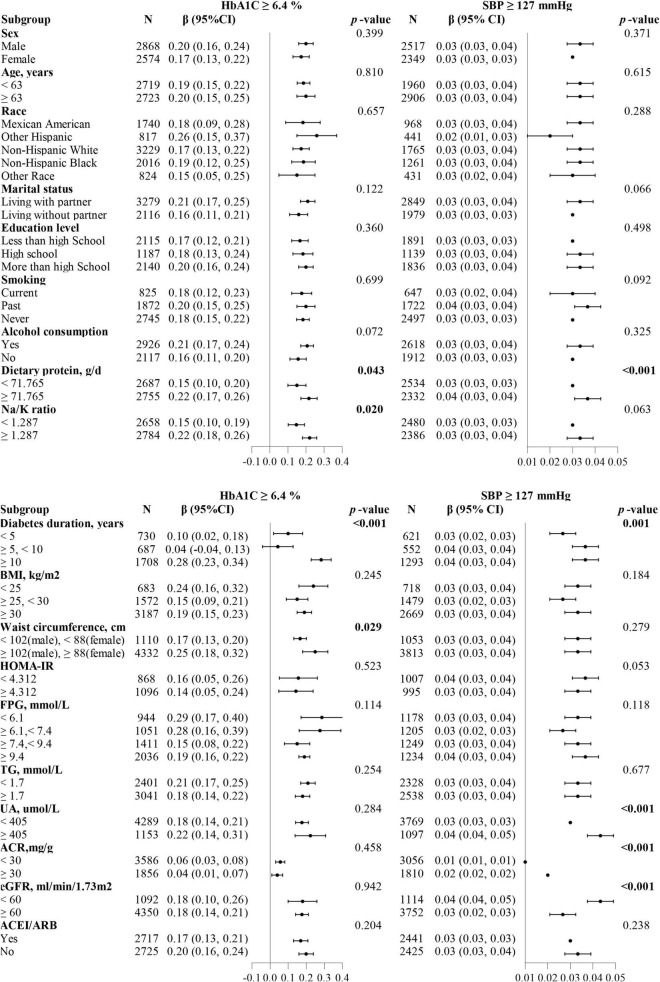
Forest plots summarizing the subgroups analyses for ACR with HbA1c or SBP divided by thresholds (HbA1c ≥ 6.4%, SBP ≥ 127 mmHg). The dietary protein, Na/K ratio, and HOMA-IR subgroups were divided based on the median. Each subgroup analysis adjusted for age, sex, marital status, education level, smoking, alcohol consumption, diabetes duration, body mass index (continuous), waist circumference (continuous), fasting plasma glucose, glycated hemoglobin/systolic blood pressure, diastolic blood pressure, triglyceride, uric acid, estimated glomerular filtration rate and dietary protein, except the subgrouping variables.

We combined discovered thresholds and explored the comprehensive effect of HbA1c and SBP levels on changes in ACR. In parallel, subgroups analyses were performed separately based on different thresholds. When the study population was divided into four groups based on two thresholds, we discovered that the dose-dependent positive relationship between the groups and the risk of elevated lnACR levels was consistently present whether adjusted for covariates (Table 4). Compared with the population who had both HbA1c and SBP levels below the thresholds, the population simultaneous above the thresholds had the fastest increase in lnACR (β = 0.67, 95 CI%:0.58, 0.76, *P* < 0.001). A rapid increase in lnACR level was more relevant to higher SBP levels above the threshold (≥127 mmHg) (Table 4). When subgroup analyses were carried out for patients with HbA1c ≥ 6.4%, significant interactions were observed both in the diabetes duration subgroup (interaction *P* < 0.001), waist circumference subgroup (interaction *P* = 0.029), dietary protein subgroup (interaction *P* = 0.043) and Na/K ratio subgroup (interaction *P* = 0.02) (Figure 3). In addition, there were also interaction effects between SBP with diabetes duration group (interaction *P* < 0.001), dietary protein subgroup (interaction *P* < 0.001), UA group (interaction *P* < 0.001), ACR group (interaction *P* < 0.001), and eGFR group (interaction *P* < 0.001) among the patients with T2DM who had a SBP level above 127 mmHg (Figure 3).

**TABLE 4:** Analysis of the combined threshold effect of both HbA1and SBP on ACR.

HbA1c (%) & SBP (mmHg)	LnACR (mg/g)						
	N	Non-adjusted model	*p*-value	Multivariate-adjusted model I	*p*-value	Multivariate-adjusted model II	*p*-value
<6.4, <127	1505	reference		reference		reference	
≥6.4, <127	2255	0.41 (0.32, 0.50)	<0.001	0.29 (0.20, 0.37)	<0.001	0.12 (0.03, 0.21)	0.006
<6.4, ≥127	1679	0.62 (0.53, 0.72)	<0.001	0.55 (0.45, 0.64)	<0.001	0.48 (0.39, 0.58)	<0.001
≥6.4, ≥127	3187	1.06 (0.98, 1.14)	<0.001	0.88 (0.80, 0.97)	<0.001	0.67 (0.58, 0.76)	<0.001

*LnACR, ln-transformed albumin/creatinine ratio; HbA1c, glycated hemoglobin; SBP, systolic blood pressure. Multivariate-Adjusted Model I adjusted for: age, sex, marital status, education level, smoking, alcohol consumption, diabetes duration, body mass index (continuous), and waist circumference (continuous). Multivariate-Adjusted Model II adjusted for: age, sex, marital status, education level, smoking, alcohol consumption, diabetes duration, body mass index (continuous), waist circumference (continuous), fasting plasma glucose, diastolic blood pressure, triglyceride, uric acid, estimated glomerular filtration rate and dietary protein.*

## Discussion

Our study elaborated on the relationship curves between ACR and HbA1c or SBP in patients with T2DM and discovered the different risk thresholds of HbA1c and SBP (HbA1c = 6.4% and SBP = 127 mmHg) above which the risk of ACR increases significantly. Additionally, more pronounced risk relationships were detected in participants with longer-duration diabetes, central obesity, or hyperuricemia.

Previously, one study discovered the threshold effect between HbA1C and ACR among a Chinese population, but it has not been studied in diabetic people (14). We first confirmed a similar association in patients with T2DM, which suggested that there exists an obvious ACR rising period that we are easy to ignore before progression to microalbuminuria. The gap between the risk threshold of HbA1c obtained in our study (6.4%) and recommended HbA1c targets (7%) (29) might be related to the early control of the above period. Notably, to define target HbA1c control levels, not only the risk of ACR progression should be taken into account, but the incidence of renal endpoints, the ultimate risk of death, and the occurrence of adverse events. Appropriate glucose control (HbA1c < 7%) recommended by the guidelines was based on a famous landmark UKPDS study (30), while the ACCORD research highlighted that intensive glucose control (HbA1c < 6%) could not reduce microvascular outcome events (31). In addition, a large-scale study with up to 13 years of follow-up reported that strict control of glucose (HbA1c < 6.5%) in the first year after newly diagnosed type 2 diabetes was associated with lower risks of diabetic vascular complications and reduced mortality (32). The aforementioned studies implied that it might be reasonable to control the HbA1c level within 6–7%, and some newly diagnosed patients would benefit more with HbA1c values < 6.5%. The threshold value (HbA1c = 6.4%) obtained in our study was also within the above range. Furthermore, a large prospective cohort study of older German adults demonstrated that increasing HbA1c (≥6.4%) was closely associated with a more than a 3-fold increased risk of decreased renal function (33). This result was generally consistent with our findings, Further, it demonstrated that there might have both short-term and long-term renal function protection when the HbA1c level was controlled below 6.4% in patients with T2DM.

As another crucial risk factor for ACR, SBP exhibited a similar threshold effect to HbA1c. However, all extensive studies emphasized the approximate range of SBP control and did not reveal the specific threshold, nor did they evaluate the risky situations under continuous changes in SBP. The existing authoritative research (34–37) results showed that patients with T2DM had a relative positive benefit-risk balance with SBP control between 120 and 140 mmHg. A prospective study on T2DM veterans discovered a significant protective benefit from lowering SBP below 130 mmHg (38), which suggested that a tighter range (120–130 mmHg) for SBP control may be required. The 127 mmHg threshold of SBP obtained in our study is also within this range. Crucially, the risk threshold detected in our study could be instrumental in the future experiment design of SBP control levels to assess long-term effects.

Combined analyses of thresholds showed the lowest ACR levels when both HbA1c and SBP control levels were below the thresholds. This was in accordance with most other studies (39, 40). Of additional concern, compared to patients with T2DM with HbA1c ≥ 6.4% and SBP < 127 mmHg, a stronger association with elevated ACR was observed in subjects with HbA1c < 6.4% and SBP ≥ 127 mmHg. These findings implied that well-controlled SBP was likely to play a more significant role in reducing urine protein levels and should be elucidated by further studies. Finally, after complete adjustment for confounding factors, the results of the subgroup analysis partially explained the heterogeneity. Our results found that longer diabetes duration and higher protein intake had interacted with HbA1c (≥6.4%) and SBP (≥127 mmHg) in the risk of ACR progression. These, too, were in keeping with previous findings. Duration of diabetes was an unmodifiable risk factor of ACR in patients with T2DM (41) while a high protein diet can exacerbate hypertension and expedite glomerular damage (42). Additionally, our results showed central obesity and a higher Na/K intake ratio could impose an extra burden on the kidney in patients with T2DM who had HbA1c ≥ 6.4%. It has been reported that central obesity could aggravate insulin resistance (43), and lead to the progression of abnormal renal hemodynamics and podocyte injury (44). The higher Na/K intake ratio might cause endothelial insult and elevate urinary protein levels (45). When SBP ≥ 127 mmHg, a more rapid rise in ACR was observed in patients with T2DM with renal insufficiency or hyperuricemia. This may be closely related to compromised kidney regulation and marked glomerular hypertension caused by the combined effects of diabetes status, hypertension, and impaired kidney function (46). Hyperuricemia is recognized as one of the risk factors for the development and progression of diabetic kidney disease. It could activate the RAAS system, further increasing blood pressure levels to promote ACR progression in patients with T2DM (47). No significant differences were identified in the subgroup analyses of age, gender, education levels, marital status, smoking and alcohol consumption, and blood lipids, which indicated that our results remain stable across most subsamples.

There are two significant clinical implications in our study. First, we identified the risk thresholds of rapid ACR progression and provided valuable references for both early blockades of DKD occurrence and development. Different from the conventional studies dividing population-based on ACR ≥ 30 mg/g to explore the potential risk factors, we described consecutive changes in ACR and observed the risk thresholds of HbA1c and SBP at an earlier level of ACR. As for patients with T2DM with normal urine protein levels, tightly controlling HbA1c and SBP within the threshold levels emphasize no proteinuria and the maintenance of long-term stable normal urine protein levels. For patients with T2DM along with proteinuria, the same control below the threshold levels may have positive significance in delaying the progression of DKD and even reversing to normal urinary protein levels (48). Second, our study further explored high-risk populations with rapid proteinuria progression, which provided partial references for individualized prevention and targeted intervention. For example, patients with T2DM diagnosed with unsatisfactory HbA1c level control should pay attention to weight management and moderately limit their protein and salt intake; patients with high SBP levels should not only reduce blood pressure reasonably but also need to check renal function regularly to prevent hyperuricemia.

Of course, our study has the following limitations. This study is cross-sectional and lacks longitudinal follow-up assessments, including primary endpoint and adverse events. More prospective studies based on our thresholds are needed in the future. In addition, it remains uncertain whether our results are generally applicable to other populations, such as Asian populations, since the enrolled participants are all from the United States.

## Conclusion

In type 2 diabetic population, we identified distinct thresholds of HbA1c and SBP (HbA1c = 6.4% and SBP = 127 mmHg) beyond which an elevated albuminuria risk would become significant. Additionally, central obesity and higher Na/K intake ratio could further increase the albuminuria risk in patients with T2DM who had HbA1c ≥ 6.4% while hyperuricemia and higher protein intake have similar effects in patients with T2DM who had SBP ≥ 127 mmHg. Our findings might have important clinical implications for the early prevention and control of DKD.

## Data Availability Statement

The original contributions presented in this study are included in the article, further inquiries can be directed to the corresponding author/s.

## Ethics Statement

The studies involving human participants were reviewed and approved by the National Center for Health Statistics (NCHS) of the Centers for Disease Control and Prevention (CDC). The patients/participants provided their written informed consent to participate in this study.

## Author Contributions

HL: conception and design. JX and YX: drafting of the manuscript and data analysis. QC and XH: reviewed/edited the manuscript. MC and JT: selection of literature and interpretation. SJ: making figures and tables. All authors contributed to the article and approved the submitted version.

## Conflict of Interest

The authors declare that the research was conducted in the absence of any commercial or financial relationships that could be construed as a potential conflict of interest.

## Publisher’s Note

All claims expressed in this article are solely those of the authors and do not necessarily represent those of their affiliated organizations, or those of the publisher, the editors and the reviewers. Any product that may be evaluated in this article, or claim that may be made by its manufacturer, is not guaranteed or endorsed by the publisher.

## Footnotes

1
http://www.r-project.org

2
www.empowerstats.net/cn/
